# Supplementary material for: Prevalence of the depression among heart failure patients in Ethiopia, 2024: A systematic review and meta-analysis
Source: PLoS One. 2025 Jun 18;20(6):e0324530. doi: 10.1371/journal.pone.0324530 (PMC12176185; doi:10.1371/journal.pone.0324530)
Supplement: S1 Table — (DOCX) [file pone.0324530.s003.docx]

**S1 Table: Search strategy of databases up to** **28^th^ November 2024**

| **PubMed** | | |
| --- | --- | --- |
| **No** | **Query** | **Records retrieved** |
| 1 | Depression | 666,786 |
| 2 | Depress | 17,941 |
| 3 | Mental disorder | 1,615,045 |
| 4 | Mood disorders | 215,615 |
| 5 | Heart Failure | 341,495 |
| 6 | Congestive Heart failure | 341,495 |
| 7 | #1 OR #2 OR #3 OR #4 | 2,027,242 |
| 8 | #5 OR #6 | 341,495 |
| 9 | #7 AND #8 | 12,530 |
| 10 | Ethiopia | 47,979 |
| 11 | Limit #9 AND #10 to English | 19 |
| **Web of science** | | |
| No | Query | Records retrieved |
| 1 | Depression | 1061448 |
| 2 | Depress | 9783 |
| 3 | Mental disorder | 574 |
| 4 | Mood disorders | 7881 |
| 5 | Heart Failure | 574654 |
| 6 | Congestive Heart failure | 4199317 |
| 7 | #1 OR #2 OR #3 OR #4 | 1703 |
| 8 | #5 OR #6 | 1426 |
| 9 | #7 AND #8 | 2471 |
| 10 | Ethiopia | 135 |
| 11 | Limit #9 AND #10 to English | 124 |
| **Google scholar** | | |
| **No** | **Query** | **Records retrieved** |
| 1 | Depression | 381 |
| 2 | Depress | 310 |
| 3 | Mental disorder | 377 |
| 4 | Mood disorders | 55 |
| 5 | Heart Failure | 2507 |
| 6 | Congestive Heart failure | 1468 |
| 7 | #1 OR #2 OR #3 OR #4 | 593 |
| 8 | #5 OR #6 | 1468 |
| 9 | #7 AND #8 | 294 |
| 10 | Ethiopia | 8,370 |
| 11 | Limit #9 AND #10 to English | 178 |
| **Scopus** | | |
| **No** | **Query** | **Records retrieved** |
| 1 | Depression | 164 |
| 2 | Depress | 13 |
| 3 | Mental disorder | 1924 |
| 4 | Mood disorders | 159 |
| 5 | Heart Failure | 294 |
| 5 | Congestive Heart failure | 257 |
| 7 | #1 OR #2 OR #3 OR #4 | 125 |
| 8 | #5 OR #6 | 82 |
| 9 | #7 AND #8 | 37 |
| 10 | Ethiopia | 26 |
| 11 | Limit #9 AND #10 to English | 15 |
| **Science Direct** | | |
| **No** | **Query** | **Records retrieved** |
| 1 | Depression | 99,271 |
| 2 | Depress | 38,289 |
| 3 | Mental disorder | 336,099 |
| 4 | Mood disorders | 127,861 |
| 5 | Heart Failure | 479,407 |
| 6 | Congestive Heart failure | 119,202 |
| 7 | #1 OR #2 OR #3 OR #4 | 98 |
| 8 | #5 OR #6 | 164 |
| 9 | #7 AND #8 | 245 |
| 10 | Ethiopia | 2,190 |
| 11 | Limit #9 AND #10 to English | 275 |
| **African journal** | | |
| No | Query | Records retrieved |
| 1 | Depression | 99,271 |
| 2 | Depress | 38,289 |
| 3 | Mental disorder | 336,099 |
| 4 | Mood disorders | 127,861 |
| 5 | Heart Failure | 479,407 |
| 6 | Congestive Heart failure | 119,202 |
| 7 | #1 OR #2 OR #3 OR #4 | 98 |
| 8 | #5 OR #6 | 164 |
| 9 | #7 AND #8 | 245 |
| 10 | Ethiopia | 2,190 |
| 11 | Limit #9 AND #10 to English | 548 |
| University repositories ( Gray literatures) | | 2 |
