# Supplementary material for: Prevalence of the depression among heart failure patients in Ethiopia, 2024: A systematic review and meta-analysis
Source: PLoS One. 2025 Jun 18;20(6):e0324530. doi: 10.1371/journal.pone.0324530 (PMC12176185; doi:10.1371/journal.pone.0324530)
Supplement: S2 Table — (DOCX) [file pone.0324530.s004.docx]

**S2 Table:** JBI Critical appraisal checklist for eligible studies

| **Studies** | **Q1** | **Q2** | **Q3** | **Q4** | **Q5** | **Q6** | **Q7** | **Q8** | **Q9** | **Overall score** | **Covert to 100%** | **Quality** |
| --- | --- | --- | --- | --- | --- | --- | --- | --- | --- | --- | --- | --- |
| Alemayehu K et al (2022) | Y | Y | Y | Y | Y | Y | N | Y | Y | 8 | 88.8 | High |
| Surafel W et al (2022) | N | Y | Y | Y | Y | Y | Y | N | Y | 7 | 77.4 | Modest |
| Afework E et al (2020) | Y | Y | Y | Y | Y | Y | Y | Y | N | 8 | 88.7 | High |
| KG Yazew et al (2019) | Y | N | Y | Y | Y | Y | Y | N | Y | 7 | 77.2 | Modest |
| Halima et al (2019) | Y | Y | Y | Y | Y | Y | Y | Y | Y | 9 | 100 | High |
| Belete et al (2019) | Y | Y | N | Y | Y | Y | Y | Y | Y | 8 | 88.1 | High |
| Henok et al (2024) | Y | Y | Y | Y | Y | Y | Y | Y | Y | 9 | 100 | High |
| Ermias et al (2024) | Y | Y | Y | N | Y | Y | Y | Y | Y | 8 | 88.3 | High |
| Tihitna et al (2024) | Y | Y | Y | Y | Y | Y | Y | Y | Y | 9 | 100 | High |
| Almaze et al (2022) | Y | Y | Y | Y | N | Y | N | Y | Y | 7 | 77.5 | Modest |
| Tegegne et al (2021) | Y | Y | Y | Y | Y | Y | Y | Y | N | 9 | 100 | High |

**Key:** Y, yes = 1; N, no = 0; JBI = Joanna Briggs Institute; overall score is calculated by counting the number of Y’s in each row.

**Notes:**

Q1 - Was the sample frame appropriate to address the target population?

Q2 - Were study participants sampled in an appropriate way?

Q3 - Was the sample size adequate?

Q4 - Were the study subjects and the setting described in detail?

Q5 - Was the data analysis conducted with sufficient coverage of the identified sample?

Q6 - Were valid methods used for the identification of the condition?

Q7 - Was the condition measured in a standard, reliable way for all participants?

Q8 - Was there appropriate statistical analysis?

Q9 - Was the response rate adequate, and if not, was the low response rate managed appropriately?
