# Supplementary material for: Prevalence of the depression among heart failure patients in Ethiopia, 2024: A systematic review and meta-analysis
Source: PLoS One. 2025 Jun 18;20(6):e0324530. doi: 10.1371/journal.pone.0324530 (PMC12176185; doi:10.1371/journal.pone.0324530)
Supplement: S3 Table — (DOCX) [file pone.0324530.s005.docx]

**S3 Table:** List of excluded full texts with reasons for exclusion

| **Author, year** | **Reason for exclusion** |
| --- | --- |
| Mulugeta H et al, 2023 [1], Belayneh M et al, 2022 [2],  Getachew A et al, 2022 [3],  Duko B et al, 2019 [4] | Different outcomes |
| Moraska AR et al, 2013 [5] Polikandrioti M et al, 2015 [6], Zahid I et al, 2018 [7]  Liu W et al, 2024 [8] | Conducted out of Ethiopia |

**References of excluded studies**

1. Mulugeta H, Sinclair PM, Wilson A (2023) Health-related quality of life and its influencing factors among people with heart failure in Ethiopia: using the revised Wilson and Cleary model. Sci Rep 13: 20241.

2. Molla B, Geletie HA (2022) Adherence to Self-Care Recommendations and Associated Factors among Adult Heart Failure Patients in West Gojjam Zone Public Hospitals, Northwest Ethiopia. 2022: 9673653.

3. Getachew A, Assefa T (2022) Self-care behavior and associated factors among patients with heart failure in public hospitals of Southeast Ethiopia. 50: 3000605221119367.

4. Duko B, Erdado M, Ebrahim J (2019) Prevalence and factors associated with depression among hospital admitted patients in South Ethiopia: cross sectional study. BMC Research Notes 12: 73.

5. Moraska AR, Chamberlain AM, Shah ND, Vickers KS, Rummans TA, et al. (2013) Depression, Healthcare Utilization, and Death in Heart Failure. Circulation: Heart Failure 6: 387-394.

6. Polikandrioti M, Goudevenos J, Michalis LK, Koutelekos J, Kyristi H, et al. (2015) Factors associated with depression and anxiety of hospitalized patients with heart failure. Hellenic J Cardiol 56: 26-35.

7. Tsabedze N, Kinsey J-LH, Mpanya D, Mogashoa V, Klug E, et al. (2021) The prevalence of depression, stress and anxiety symptoms in patients with chronic heart failure. International Journal of Mental Health Systems 15: 44.

8. Liu W, Lin Q, Fan Z, Cui J, Wu Y (2024) Major depression disorder and heart failure: A two-sample bidirectional Mendelian randomization study. 19: e0304379.
