# Supplementary material for: Prevalence of the depression among heart failure patients in Ethiopia, 2024: A systematic review and meta-analysis
Source: PLoS One. 2025 Jun 18;20(6):e0324530. doi: 10.1371/journal.pone.0324530 (PMC12176185; doi:10.1371/journal.pone.0324530)
Supplement: Attachment — Submitted filename: My review to plos one.docx [file pone.0324530.s007.docx]

**Dear Editor and author,**

I would like to extend my heartfelt thanks to Addisalem Workie, the academic editor of PLOS ONE, for inviting me to review the manuscript titled "***A Systematic Review of Depression among Heart Failure Patients in Ethiopia, 2023***" for PLOS ONE. It was an honor to be entrusted with this task.

Reviewing this manuscript was an enriching experience. The research fills a crucial gap in the literature and addresses a significant public health issue in Ethiopia. I commend the authors for their meticulous work, and I also commend the editorial team for recognizing its importance.

Thank you once again for this opportunity to contribute to the advancement of knowledge in this field. I appreciate the chance to engage with this important topic. However, I have identified several limitations within the study that need to be addressed before I can recommend acceptance for publication. Please carefully review the comments I have provided below:

1. Your topic is interesting, but there are already two papers published on it, both systematic reviews and meta-analyses. What makes your paper unique? If there are differences, please clearly state them to avoid redundancy. Please refer to the following publications and respond accordingly:
a. Mulugeta et al. [Prevalence of **depression** and its association with health-related quality of life in people with **heart** **failure** in low- and middle-income countries: A **systematic** **review** and meta-analysis.](https://pubmed.ncbi.nlm.nih.gov/36952483/)
b. Firomsa et al. [Adherence to self-care practices and associated factors among **heart** **failure** patients in **Ethiopia**: A **systematic** **review** and meta-analysis.](https://pubmed.ncbi.nlm.nih.gov/37611019/)
2. Regarding data availability, in your online submission, you stated/selected that "all data are fully available without restriction," but in your online submission, you wrote "Data will be available upon request of corresponding author." Please ensure consistency in your statements.

In the Methods section of your abstract, you mentioned, "Analysis was conducted using STATA version 11 software, with assessment of heterogeneity and publication bias. A random-effects meta-analysis model was used to estimate the pooled prevalence of depression." However, I could not find the assessment of heterogeneity and publication bias in the manuscript. Additionally, you uploaded the subgroup analysis of heterogeneity without discussing them in the Results section. Please revise your Results section to include all subgroup analysis results and their respective values. If there is heterogeneity, you have to conduct Univariate Meta-Regression and report your findings. Based on those results, you need to separately present your subgroup analysis findings.

3. Where is the statistical measure of your publication bias?
4. Under the Results section, there are no graphical representations of the included studies. Please update your manuscript to include appropriate tables, figures, or any other illustrative formats.
5. Your results section lacks chronological order, coherence, or may be missing information. Please revise it for clarity and organization.
6. There is evidence of copywriting or plagiarism
7. Your discussion section is lacking in depth and clarity. Please work on making it understandable, well-discussed, justified, and engaging.
8. Avoid using abbreviations in captions, titles, or abstracts.
9. Where are your recommendations??
10. Modify figure 1.
11. Your declaration section lacks coherence and completeness. For instance:
i. Your statement "Ethics approval and consent to participate, Acknowledgements: Not applicable" requires clarification. Why is it not applicable?
ii. Regarding the availability of data and materials: "All available data are within the paper" suggests full accessibility without restrictions, while "Data will be available upon request of the corresponding author" implies a different level of accessibility. There is inconsistency regarding data availability in both your manuscript and the online submission.
