# Supplementary material for: Prevalence of the depression among heart failure patients in Ethiopia, 2024: A systematic review and meta-analysis
Source: PLoS One. 2025 Jun 18;20(6):e0324530. doi: 10.1371/journal.pone.0324530 (PMC12176185; doi:10.1371/journal.pone.0324530)
Supplement: Attachment — Submitted filename: Authors response to Reviewers #Review1.docx [file pone.0324530.s008.docx]

| **Authors’ point-by-point response to the editor and reviwers report**  **Prevalence of the depression among Heart failure patients in Ethiopia, 2024: A Systematic Review**  Birhaneslasie Gebeyehu Yazew^1^*, Workineh Tamir Shitu^2‡^, Mohammed Hassen Salih^3‡^, Zewdu Bishaw Aynalem^1‡^, Haile Workye Agazhu^1‡^, Daniel Adane Endalew^4‡^ | | | |
| --- | --- | --- | --- |
| **Editor#1** | **Original comments of the editor** | **Reply by the author(s)** | **Changes done on page number and line number** |
| **1** | The database search was conducted in March 2023, but the PROSPERO protocol has not been registered until now, which is a significant limitation. | Dear editor, thank you for your comment and time!  **Reply:** we accept and add the given PROSPERO protocol number CRD42023405077.  <https://www.crd.york.ac.uk/prospero/export_details_pdf.php> | See the track change document page 5 line number 101-104. |
| **2** | Since that search, relevant papers have been published on this topic and should be included in the analysis. | Dear editor, thank you for your comment and time!  **Reply:** We have researched upto 28^th^ November, 2024 and reanalyze by incorporating additional 5 new papers. | See the track change document page 6, line number 117 & 118, page 10, line number 204-212, and table 1. |
| **3** | You conducted  a meta-analysis of proportions using STATA version 11. However, this analysis type was introduced in late 2023, and while some researchers provided a (metaprop) command for this analysis in 2014, it only functioned on STATA version 14 or later. I am curious about how the authors managed to perform it with version 11. | Dear editor, thank you for your constructive comment!  **Reply:** Accept and reanalyze using STATA version 17 by adding additional 5 papers. | See the track change document, page 2, line number 32, page 8 line number 151 and 155. |
| **4** | Check your PRISMA flow chart. It should be updated and recent one | Dear editor, thank you for your comment!  **Reply:** accept and updated. | See the track change document page 9, line number 188-195 and figure 1. |
| **Reviewer#1** |  |  |  |
| **1.1** | Your topic is interesting, but there are already two papers published on it, both systematic reviews and meta-analyses. What makes your paper unique? If there are differences, please clearly state them to avoid redundancy. Please refer to the following publications and respond accordingly: a. Mulugeta et al. Prevalence of depression and its association with health-related quality of life in people with heart failure in low- and middle-income countries: A systematic review and meta-analysis. b. Firomsa et al. Adherence to self-care practices and associated factors among heart failure patients in Ethiopia: A systematic review and meta-analysis. | Dear reviewer, thank you for your constructive comment!  **Reply:-** Infact, we have seen those published works. However, the following gaps or reasons are lead us to research on. 1^st^. Mulugeta and his colleagus were focused on low- and middle-income countries, and also no any pooled prevalence of depression that showed in Ethiopia.  2^nd^. Firmosa’s and his colleagus work was also focussed on adherence on Self-care Behaviour, which their objectives was not related to depression. Hence, estimating pooled depression in Ethiopia is unique and researchable. |  |
| **1.2** | Regarding data availability, in your online submission, you stated/selected that "all data are fully available without restriction," but in your online submission, you wrote "Data will be available upon request of corresponding author." Please ensure consistency in your statements | Dear reviewer, thank you for your comment!  **Repley**:- We accepte and correct it. | See the online submission. |
| **1.3** | In the Methods section of your abstract, you mentioned, "Analysis was conducted using STATA version 11 software, with assessment of heterogeneity and publication bias. A random-effects meta-analysis model was used to estimate the pooled prevalence of depression." However, I could not find the assessment of heterogeneity and publication bias in the manuscript. | Dear reviewer, thank you for your comments!  **Repley**:- We accept and make modification accordingly. Firstly, we add 5 additional articles by researching until 28^th^ November, 2024. Secondly, we reanalyze using using STATA version 17. Thirdly, we put the assessment of heteroginity and publication bais in the manuscript. | See the track change  of the document page 14 up to 15, line number 265-300. |
| **1.4** | Additionally, you uploaded the subgroup analysis of heterogeneity without discussing them in the Results section. Please revise your Results section to include all subgroup analysis results and their respective values. | Dear reviewer, thank you for your comments!  **Reply:** We revise and include all respective values in result section. | See the track change  of the document page 2 upto 3, line number 36-44, page 12 upto 13, line number 233--257. |
| **1.5** | If there is heterogeneity, you have to conduct Univariate Meta-Regression and report your findings. Based on those results, you need to separately present your subgroup analysis findings. | Dear reviewer, thank you for your comments!  **Reply:** We accept and conduct it. | See the track change  of the document page 14-15, line number 282-297, and table 2. |
| **1.6** | Where is the statistical measure of your publication bias? | Dear reviewer, thank you for your comments!  **Repley**:- We reanalyze and measure it. | See the track change document at the page 15-16, line number 299-313, and table 3. |
| **1.7** | Under the Results section, there are no graphical representations of the included studies. Please update your manuscript to include appropriate tables, figures, or any other illustrative formats. | Dear reviewer, thank you for your comments!  **Repley**:- We accept and update it. | See the track change document at the page 10-11, line nimber 208-218, figure 1, and table 1. |
| **1.8** | Your results section lacks chronological order, coherence, or may be missing information. Please revise it for clarity and organization. | Dear reviewer, cordially thank you for your constructive comments!  **Repley**:- We accept and revise accordingly. | See the track change document page 9 upto 17, line number 189-320. |
| **1.9** | There is evidence of copywriting or plagiarism | Dear reviewer, thank you for your comments!  **Repley**:- This is our orginal work, however it may have the similarity of the words. Thus, we accept and reparaphrases it through out the document. | See the track change throughout the document. |
| **1.10** | Your discussion section is lacking in depth and clarity. Please work on making it understandable, well-discussed, justified, and engaging. | Dear reviewer, thank you for your comments!  **Repley**:-Accept and we made it in understandable manner. | See the track change document page 17, line number 325-329, page 17 line nimber 334-338, and page 18, line numbe 345-359. |
| **1.11** | Avoid using abbreviations in captions, titles, or abstracts. | Dear reviewer, thank you for your comments!  **Repley**:-we accept and avoid it. | See the track change throughout the document. |
| **1.12** | Where are your recommendations?? | Dear reviewer, thank you for your comments!  **Repley**:-we accept and make an amendment by adding conclusion and recommendations at the heading. | See the track change document page 19, line number 369-376. |
| **1.13** | Modify figure 1. | Dear reviewer, thank you for your comments!  **Repley**:-we accept and modify it. | See the track change document at Figure 1. |
| **1.14** | Your declaration section lacks coherence and completeness. For instance:  Your statement "Ethics approval and consent to participate, Acknowledgements: Not applicable" requires clarification. Why is it not applicable? | Dear reviewer, thank you for your constructive comments!  **Repley**:- If it is registered at PROSPERO, Ethical clearance is not applicaple because this study was based on already published primary studies.  Whereas regrading the **acknowledgment**, we accept your comment and add as follows….’we would like to acknowledge all the authors of the original studies included in this systematic review’. Thus, we accept and edited it. | See the track change document page 20, line number 390 -398. |
| **1.15** | Regarding the availability of data and materials: "All available data are within the paper" suggests full accessibility without restrictions, while "Data will be available upon request of the corresponding author" implies a different level of accessibility. There is inconsistency regarding data availability in both your manuscript and the online submission. | Dear reviewer, thank you for your comments!  **Repley**:- we accept and make consistant by replacing the online’s idea with ‘All available data are within the paper’. | See the submitted document on the online. |
| **Reviewer#2** |  |  |  |
| **2.1** | I would like to thank the authors for their efforts in analyzing the prevalence of depression among HF patients in Ethiopia. Unfortunately, given the numerous limitations in the manuscript, my response cannot be more positive. | Thank you for your idea dear reviewer.  **Reply:-** We accept and respect your idea. |  |
| **2.2** | Database search  The database search has been done in March 2023 and yet the PROSPERO protocol was not registered thus far. It means that you had it registered post-hoc, which is a major drawback. | Dear reviewer, thank you for your constructive comments and time!  **Reply:** We accept and add the given PROSPERO protocol number CRD42023405077.  <https://www.crd.york.ac.uk/prospero/export_details_pdf.php> | See the track change document page 5 line number 103-105. |
| **2.3** | Since March 2023, 2-3 relevant papers have been published on this topic that should be included in the analysis. I assume more would be found on other database (particularly Google Scholar). You need an updated search. | Dear reviewer, thank you for your comment and time!  **Reply:** We have researched up to 28^th^ November, 2024 and reanalyze by incorporating additional 5 new searched papers. | See the track change document page 6, line number 118-123, page 9-10 line number 189-206, table 1, Figure 1, and S1 table. |
| **2.4** | I am assuming there is a problem with the syntax you used for the database search because getting only 6 studies out of 150631 screened articles outlines a HUGE Problem, either with the syntax or with the screening. You would need to provide the detailed search query employed for each database. Also, please be advised that only the 1st 200 records from Google Scholar are to be included (as per guidelines). | Dear reviewer, we appreciate and thank you for constructive comments!  **Reply:-** We accept and provide the search query that we have researched by accepting the comments given from you at the above comment number 2.3 and from other reviewers. By including the first 200 records from Google scholar, we got screened 11 studies out of the 1159 retrived articles. | See the track change document at the 6, line number 118-123, page 9 line number 189-206, table 1, Figure 1, and S1 table. |
| **2.5** | Methodology: 1- Having 6 authors do a review of 6 papers is a bit confusing to me. | Dear reviewer, we respect your idea!  **Reply:-** Since this is a team work, each authors must contribute their potential for better outcome. Thus, authors found in this study had contributed in different tasks equally. | See the track change document at the page 8, line number 163-166. |
| **2.6** | 2- You have not outlined how depression and HF were diagnosed in each study. Maybe there could be a difference based on the diagnostic criteria. | Dear reviewer, we appreciate your comment and thank you!  **Reply:-** we accept and outlined diagnosis of depression and HF or their tools of measurement that was used in primary studies. However, we didn’t perform subgroup analysis by tool. Because, of the 11 studies, 10 were conducted with simmiler tool PHQ-9 and only 1 study was also used BDI. Hence, it is not reccomendale to perform subgroup analysis by tool in this occassions. | See the track change document page 7, line number 135-144. |
| **2.7** | 3- Again, the limited sample size makes it very difficult to delve into the (effect modifying role) of these paramters (i.e., diagnositc criteria, sampling method, etc.) because you need to have at least 3 studies in each subgroup to provide interpretable evidence; otherwise, it would be too inaccurate. | Dear reviewer, we appreciate your constructive comments and thank you!  **Reply:-** Exactly! After accepting yours and the other reviewers comment, we have reanalyzed it by adding additional 5 articles and tray to minimize this gap. However, still there is some gap and we had stated it at the limitations of the study. | See the track change document page 20, line number 381-389. |
| **2.8** | 4- You did not report the severity of depression +/- HF in the analysis. | Dear reviewer, thanky for your constructive comment and raised issues!  **Reply:** We accept and reported. | See the track change document page 12, line number 225-228. |
| **2.9** | 5- The English structure of the paper is very poor. It needs professional editing. | Dear reviewer, thanky for comment!  **Reply:** We accept and gave the manuscript for the linguistic professional found at the Department of English and Litrature, Injibara University and he edited spelling, grammer, and others accordingly. | See the track change throughout of the document. |
|  | **Statistical analysis** |  |  |
| **2.10** | 1- The authors performed meta-analysis of proportion on STATA version 11. This type of analysis was introduced in late 2023 to STATA. However, some authors provided a (metaprop) command to carry out this analysis in 2014. At that time, this command would only run on STATA version 14 or beyond. I am wondering how the authors performed it using version 11. | Thank you for comment!  **Reply:** We accept and perform reanalysis after adding additional 5 articles by using STATA 17. | See the track change document page 2, line number 31 and page 8, line number 168, 172, and 173. |
| **2.11** | Publication bias assessments needs at least 10 studies to make (powered analysis) through Funnel plot. Runnign a Funnel plot with 6 studies is not powered to detect any publication bias. | Dera reviewer, thank you for comment!  **Reply:** We extend the searching time up to 28^th^ November, 2024 and got additional 5 articles and perform re-assessment of publication bais. | See the track change document page 6, line number 118 & 119, page 15-6, line number 299-313 and Figure 6. |
| **2.12** | Added value: 1- There is an already published MA on this topic which provides not only region-based prevalence of Depression in HF, but also its imapct on quality of life. The paper is already published in PLOS ONE (<https://pmc.ncbi.nlm.nih.gov/articles/PMC10035817/>) | Dear reviewer, thank you for your constructive comment!  **Reply:-** Actually, we have seen this published work. However, the following reason which leads us to research on. Mulugeta and his colleagus were focused on low- and middle-income countries, and even Ethiopia was includeded in their study, no any pooled prevalence of depression that showed specifically for Ethiopia. Therefor, we conducted this review to answer this question related to estimation of pooled depression. |  |
| **Review#3** |  |  |  |
| **3.1** | THE TITLE o The title is very narrow I suggest the authors to improve and make it to sound scientific manner. | Thank you for comment!  **Reply:-** We accept and improved. | See the track change document page 1, line number 1-3. |
| **3.2** | ABSTRACT METHODS o I noted the authors didn’t address the study durations also inclusions criteria of the study. | Thank you for comment!  **Reply:-** We accept and addressed. | See the track change document page 2, line number 29 - 30. |
| **3.3** | RESULT o On part of result I noted the authors didn’t explain some of information which is very important. I suggest the authors to revise and improve on it. | Thank you for comment!  **Reply:** we accept and improve it. | See the track change document page 2 & 3, line number 36-44. |
| **3.4** | CONCLUSIONS o The author should I make the conclusions according to the result | Dear reviewer, thank you for comment!  **Reply:** accept and made an amendment. | See the track change document page 3, line number 47-51. |
| **3.5** | INTRODUCTIONS o Very clear and understanding but I noted the authors should correct at the last paragraph of introductions. To the best of our knowledge, there is no existing systematic review on the prevalence of depressive symptoms among heart failure patients in Ethiopia. Understanding and estimating depression among heart failure patients can aid in enhancing the health status of individuals by addressing the negative impact of depression and other complications related to heart failure. Therefore, this systematic review aims to consolidate data on the prevalence of depressive symptoms among heart failure patients in Ethiopia. I suggest the authors should use the scientific language. | Dear reviewer, thank you for comment!  **Reply:** We accept and make correction. And also, made language edition by professional found at the Department of English and Litrature, Injibara University throughout the document. | See the track change document page 4-5, line number 80-99. |
| **3. 5** | o I would ask the authors why the review is necessary gaps in existing of knowledge. I suggest make clear justifications of your review. | Dear reviewer, we would like cordially thank you for a such knowledge builder comments!  **Reply:-** Because, to determine the pooled prevalence of depression among heart failure patients. Thus, having this estimated depression among HF patients in Ethiopia is also will help: for policy makers and program designers to plan the appropriate interventions, for health care workers to work on rehabilitations, and for researchers to see unexplored areas of inquiry and avoid duplication. | See the track change document page 5, line number 96-99. |
| **3.6** | METHODS o On part of study protocol and registrations I suggest this is systematic review manuscript so if it has no registrations number it won’t be publish its better to have them ready. | Dear editor, thank you for your comment and time!  **Reply:** we accept and add the given PROSPERO protocol number CRD42023405077.  <https://www.crd.york.ac.uk/prospero/export_details_pdf.php> | See the track change document page 5 line number 103-105. |
| **3. 7** | o I noted the authors written this A systematic literature search had made by two investigators (BG and YB) what does it meaning? I suggest to remove are not fit on this part already classify on another part. | Thank you for a constructive comment!  **Reply:** We accept and removed it. | See the track change document page 5, line number 107. |
| **3.8** | o Also the authors should revise on this part there are some error appeared like (((((Depression [Mesh Terms]) OR (depress*[Mesh Terms])) OR (“Mental disorder” [Text Word])) OR (“Mood disorder” [Text Word])) AND ((Heart failure) OR (congestive heart failure [Mesh Terms])) I suggest improve them and make it clear. | Dear reviewer, cordially thank you for a constructive comment!  **Reply:** We accept and correct it. | See the track change document page 6, line number 12 -115. |
| **3.9** | Outcome measure  o The authors should explain each one and their procedure. | Dear reviewer, cordially thank you for showing us missed figure to attach!  **Reply**: We accept and explain each. | See the track change document page 7, line number 135-144. |
| **3.10** | o Where is the diagram on part of study [selection. it](http://selection.it/) should be included on your document | Thank you for a good comment!  **Reply:** we accept and included. | See the track change document page 7-8, line number 154-157, and S2 table. |
| **3.11** | o On this part the authors should be revise are not clear some of information is confusing when reading. | Dear reviewer, we really appreciate and cordially thank you for such constructive comments!  **Reply:** We accept and revised the statement of the authors contribution. | See the track change document page 8, line number 163-166. |
| **3.12** | o TABLE 1 I noted the authors should revise because the way the authors present the heading above is not clear. | Thank you for a comment!  **Reply:** We accept and revised. | See the track change document page 10 & 11, table 1. |
| **3.13** | DISCUSSIONS o In the part of discussion I noted is very narrow there is no comparison of the findings align or contract with prior research the authors should revise and improve them. | Dear reviewer, thank you for your comments!  **Repley**:-Accept and we revise it. | See the track change document page 17, line number 325-329, page 17-18, line numbe 334-338, and page 18, line number 345-359. |
| **3.14** | o What is your strength on your systematic review? | Dear reviewer, thank you for question and a comment!  **Reply:** This review has provided valuable information on the estimated prevalence of depression among HF patients by using different databases to search the articles and by using six reviewers to minimize reviewers’ bias and quality evaluation | See the track change document page 19, line number 378-380. |
| **3.15** | LIMITATIONS o Also there are no limitations concerning to your study such as potential bias limitations in the available data and study limitations. I suggest the authors should revise and improve on it. | Thank you for a productive comment!  **Reply:** we accept and add it. | See the track change document page 20, line number 381-389. |
| **3.16** | Declaration o I adverse the authors to remove this part and follow PLOSE ONE guideline | Thank you very much for agood comment!  **Reply:** accept and remove it. | See the track change document page 20, lin number 390 |
| **3.17** | NB: o The authors should consider the spelling errors when writing the manuscript also the authors should revise all documents and use the high Standard English which is used in research. Because this paper are very interesting. | Thank you for a good comment!  **Reply:** We accept and mad language edition by providing it for the professional found at the Department of English and Litrature, Injibara University. | See the track change throughout of the document. |
|  | **Again we would like to say thank you for all given comments and your time!** | |  |
